# Poland’s syndrome with dextroposition and paradoxical breathing

**DOI:** 10.11604/pamj.2021.38.110.27384

**Published:** 2021-02-03

**Authors:** Ng Kian Seng, Sarvesh Seger

**Affiliations:** 1 Department of Internal Medicine, International Medical University, Clinical Campus Kluang, Kluang, Johor, Malaysia,; 2 School of Medicine, International Medical University, Clinical Campus Kluang, Kluang, Johor, Malaysia

**Keywords:** Poland´s syndrome, dextroposition, cervical ribs, electrocardiogram, chest radiograph

## Image in medicine

An asymptomatic 60-year-old woman presented to our outpatient clinic for a routine health screening as part of a government welfare program. Her medical history was unremarkable, however physical examination revealed hypoplasia of the left chest wall muscles, breast tissue, nipple, absence of axillary hair, absence of the costal cartilages of upper ribs on the left side of the chest and brachydactyly in the left hand. There was also paradoxical breathing at the levels of the 2^nd^, 3^rd^ and 4^th^ rib of the left chest. The rest of the examination was unremarkable. The chest radiograph revealed reduced upper left hemithorax volume with partial rib agenesis of the 2^nd^, 3^rd^ and 4^th^ rib, bilateral cervical ribs, dextroposition of the heart and mid thoracic scoliosis with convexity to the left. A 12-lead electrocardiogram revealed a sinus rhythm with a normal axis, lead 1 has upright P, QRS, and T waves and aVR is globally negative. The precordial leads show normal R Waves in V1-4, deep S waves in V1-2, small S waves in V3-4 and no S waves in V5-6. There is diminution of the precordial complexes in V3, 4, 5 and 6. These findings are consistent with a dextroposition of the heart. The blood investigations were unremarkable. The findings from physical examination, chest radiograph and 12 lead electrocardiogram are consistent with Poland´s Syndrome. As the patient was otherwise well and not bothered by her condition, no treatment was initiated for her.

**Figure 1:**
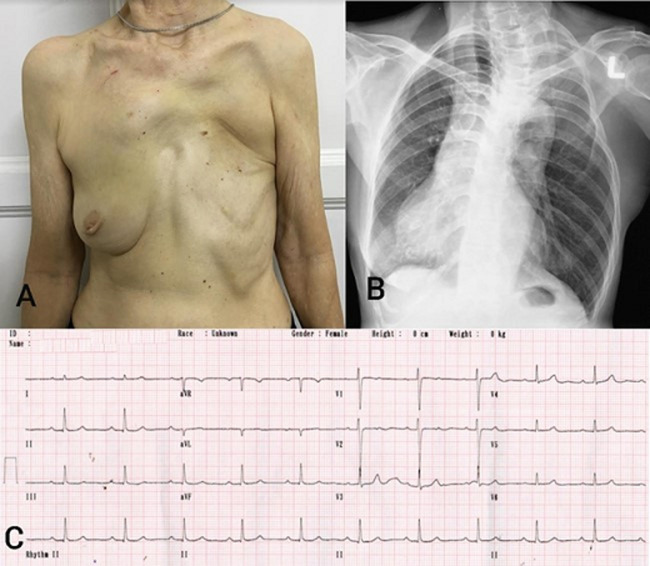
A) photograph of patient’s chest with Poland’s syndrome; B) chest radiograph of the patient; C) 12-lead electrocardiogram of the patient

